# Thy-1-Induced Migration Inhibition in Vascular Endothelial Cells through Reducing the RhoA Activity

**DOI:** 10.1371/journal.pone.0061506

**Published:** 2013-04-17

**Authors:** Heng-Ching Wen, Chieh Kao, Ruei-Chi Hsu, Yen-Nien Huo, Pei-Ching Ting, Li-Ching Chen, Sung-Po Hsu, Shu-Hui Juan, Wen-Sen Lee

**Affiliations:** 1 Graduate Institute of Medical Sciences, Medical College, Taipei Medical University, Taipei, Taiwan; 2 Graduate Institute of Cell and Molecular Biology, Medical College, Taipei Medical University, Taipei, Taiwan; 3 Department of Physiology, School of Medicine, Medical College, Taipei Medical University, Taipei, Taiwan; 4 Cancer Research Center, Taipei Medical University Hospital, Taipei, Taiwan; UAE University, United Arab Emirates

## Abstract

Our previous study indicated that Thy-1, which is expressed on blood vessel endothelium in settings of pathological and a specific of physiological, but not during embryonic, angiogenesis, may be used as a marker for angiogenesis. However, the function of Thy-1 during angiogenesis is still not clear. Here, we demonstrate that knock-down of the endogenous Thy-1 expression by Thy-1 siRNA transfection promoted the migration of human umbilical vein endothelial cells (HUVEC). In contrast, treatment with interleukin-1β (IL-1β) or phorbol-12-myristate-13-acetate (PMA) increased the level of Thy-1 protein and reduced the migration of HUVEC. These effects were abolished by pre-transfection of HUVEC with Thy-1 siRNA to knock-down the expression of Thy-1. Moreover, over-expression of Thy-1 by transfection of HUVEC with Thy-1 pcDNA3.1 decreased the activity of RhoA and Rac-1 and inhibited the adhesion, migration and capillary-like tube formation of these cells. These effects were prevented by co-transfection of the cell with constitutively active RhoA construct (RhoA V14). On the other hand, pre-treatment with a ROCK (a kinase associated with RhoA for transducing RhoA signaling) inhibitor, Y27632, abolished the RhoA V14-induced prevention effect on the Thy-1-induced inhibition of endothelial cell migration and tube formation. Taken together, these results indicate that suppression of the RhoA-mediated pathway might participate in the Thy-1-induced migration inhibition in HUVEC. In the present study, we uncover a completely novel role of Thy-1 in endothelial cell behaviors.

## Introduction

Angiogenesis, the generation of new blood vessels from pre-existing ones, occurs during embryonic development, body growth, formation of the corpus luteum and endometrium, tissue regeneration and wound healing [1]. Abnormal angiogenesis also plays an important role in many pathological processes, including tumor growth, metastasis, diabetic retinopathy and arthritis [2], [3]. Angiogenesis is a complex multi-step process involving extensive interplay between cells, soluble factors, and extracellular matrix (ECM) components. Only when the process and regulation of angiogenesis is fully understood can we begin to design a strategy for treating angiogenesis-related disorders.

In the angiogenic process, migration and proliferation of endothelial cells are two critical steps. Angiogenesis is under tight control by a balance of angiogenesis inducers and inhibitors. Activated endothelial cells reorganize their cytoskeletons, express cell surface adhesion molecules such as integrins and selectins, secrete proteolytic enzymes, and remodel their adjacent ECM [2]. Dynamic interactions between cell surface adhesive receptors (integrins) for ECM components, organization of the actin cytoskeleton and the turnover of focal adhesions are all key processes in cell locomotion and migration. Although the mechanism of angiogenesis regulation is not fully elucidated, it is generally accepted that the initiation or termination of the process is controlled by a balance between positive and negative regulators of angiogenesis. Angiogenesis may occur in the organism as a result of a number of different stimuli such as vascular injury, wounds, neoplastic growth, and/or local inflammation. While the initiation of angiogenesis has been intensive studies, little is known about the control of termination of angiogenesis. Understanding the molecular mechanism of termination of angiogenesis might provide novel strategies for therapeutic intervention of vascular growth.

Previously, we observed that Thy-1 serves as a marker for angiogenesis and demonstrated that Thy-1 was expressed during physiological and pathological angiogenesis in adult rats, but not during embryonic angiogenesis [4]. Thy-1, a 25–37 kDa glycosylphosphatidylinositol (GPI)-anchored cell surface protein, belongs to the immunoglobulin-like supergene family. Originally, Thy-1 was described as a marker for thymocyte differentiation in mice. Subsequently, Thy-1 was found to be expressed in various cell types, including neurons, fibroblasts, ovarian cancer cells, hematopoietic cells and vascular endothelial cells [5], [6]. Although Thy-1 has been suggested to be involved in cellular growth, differentiation, apoptosis, adhesion, and migration, the function of Thy-1 expression during angiogenesis is still unknown. Previous in vitro studies have shown that upon stimulation such as phorbol-12-myristate-13-acetate (PMA) as well as inflammatory cytokines, interleukin-1β (IL-1β) or tumor necrosis factor-α (TNF-α), up-regulated Thy-1 mRNA in vascular endothelial cells. In the present study, we investigated the possible role of Thy-1 during angiogenesis by examining the relationship between the Thy-1 level in vascular endothelial cells and endothelial cell proliferation and migration. We found that up-regulation of Thy-1 expression by treatment of HUVEC with IL-1β or TNF-α or transfection with Thy-1 pcDNA3.1 inhibited, while knock-down the endogenous Thy-1 expression promoted, the migration in cultured human vascular endothelial cells.

## Materials and Methods

### Cell Culture

HUVEC or an immortalized human microvascular endothelial cell line (HMEC-1) [7] were grown in M199 (GIBCO, Grand Island, NY) containing 10% FBS (Highveld Biological, Lyndhurst, RSA), endothelial cell growth supplement (ECGS, 0.03 mg mL^−1^) (Biomedical Technologies, Stoughton, MA) and kanamycin (GIBCO) (50 U mL^−1^) in a humidified 37°C incubator. Cells from passages 5–10 were used.

### Plasmids

Human Thy-1 cDNA was obtained from EST clone and cloned into Lamfmid vector via the cloning sites of Hind III and Not I. For over-expression of human Thy-1, the full length of Thy-1 was subcloned to the expression vector, pcDNA3.1(+) (Invitrogen) at the same restriction sites as noted above and the expression is driven by the CMV promoter.

### Cell Transfection

For transient transfection of the Thy-1 or constitutively active RhoA (RhoA V14) constructs into HUVEC, jetPEI-HUVEC transfection reagent (Polyplus Transfection, Bioparc, France) was used according to the manufacturer’s protocol. Briefly, a jetPEI-HUVEC/DNA mixture was added drop-wise onto the M199M+Glutamax™ I medium (GIBCO) containing 2% FBS, mixed gently, and incubated in a humidified 37°C incubator for 4 h. The growth medium was then replaced and the cells were incubated further for 24 h. To verify the transfection efficiency, HUVEC was transfected with Thy-1-pCMS-EGFP (enhanced green fluorescent protein)-C1, and then monitored using an inverted fluorescent microscope.

### Adhesion Assay

HUVEC or HMEC-1 transfected with vector (pcDNA3.1) or Thy-1 pcDNA3.1 were plated onto a collagen (0.1 mg/mL)-coated 24-well plate for various as indicated at 37°C, and then washed with phosphated-buffered saline (PBS). After washing, the number of adherent cells was assessed by MTT (3-[4,5-dimethylthiazol-2-yl]2,5-diphenyltetrazolium bromide) assay [8] or cell count. For MTT assay, the cell was incubated with various time periods, and tetrazolium-based compound (MTT) was added into individual wells at a final concentration of 125 µg/mL. After incubation at 37 C for 4 h, the media were removed, and 100 µL DMSO was added to dissolve the formazan dye. The purple formazan solution was transferred to 96-well plates and immediately read at 540 nm with a spectrophotometer (BioTek-uQuant, Winooski, VT, USA). For cell count, the cell was incubated for 14 h, washed with fresh medium to remove the non-attached cells, and then the cell number attached onto a collagen-coated 12-well plate was counted under a light microscope.

### RT-PCR

Total cellular RNA was extracted from HUVEC with Trizol (Gibco) according to the manufacturer’s protocol [9]. Two µL of total RNA were used in a total of 20 µL reaction volume as a template for PCR amplification. PCR was performed under standard conditions in 20 µL of 10 mM Tris, pH 8.3, 40 mM KCl, 1.5 mM MgCl_2_, 250 µM dNTP, 10 pM of each primer (sense and antisense) and 1 U Taq DNA polymerase. The experimental conditions were as follows: 95°C for 1 min; 55°C for 1 min; and 72°C for 1 min. The PCR regimen was: 5′-ATGAACCTGGCCATCAGCAT-3′, 5′-TCACAGGGACATAAAATCCGTG-3′ for Thy-1; 5′-TGAAGGTCGGAGTCAACGGATTTGGT-3′, 5′-CATGTGGGCCATGAGGTCCACCAC-3′ for G3PDH [10]. The PCR products were electrophoresed on a 1.8% agarose in 1 × Trisacetate-EDTA (TAE) buffer, and stained with ethidium bromide solution.

### Western Blot Analysis

HUVEC were cultured in 10 cm petri dishes. After reaching subconfluence, the cells were tranfected with vector (pcDNA3.1) or Thy-1 cDNA and then grown in the culture medium containing 10% FBS at 37°C. At 24 h after transfection, the cells were processed for protein extraction and Western blot analysis as previously described [11].

### RhoA Activity Assay

The RhoA activity was measured by affinity precipitation of active (GTP-bound) RhoA from cell lysates (500 µg) using Rho activation assay kit (Millipore, Temecula, CA) following manufacturer’s protocol.

### Cell Invasion Assay

The cell invasion assay was performed as described previously with minor modifications [12]. To assess the migration potential of HUVEC, the lower face of Transwells (8 µm pore size) was precoated with Type-1 collagen at a concentration of 1 mg/mL for 1 h at 37°C. The Transwells were then assembled in a 24-well plate, and the lower chambers were filled with 600 µL of M199 containing 10% FBS and endothelial cell growth stimulating factors. Two hundred µL of cells (10^5^ cells/mL) were inoculated onto the upper chamber of each Transwells. The plate was then placed at 37°C in 5% CO_2_/95% air for 18 h. After removing the non-migrating cells with a cotton swab, cells that had migrated to the lower surface of the filters were fixed and stained with 0.1% crystal violet/20% (v/v) methanol. All assays were performed in triplicate. Three random fields were chosen in each insert, and the cells were counted and photographed under a light microscope (×200).

### Lamellipodia Assay

HUVEC transfected with pcDNA3.1 or Thy-1 pcDNA3.1 were seeded on coverslips and incubated in growth medium for 40 h, fixed in 4% paraformaldehyde, and then permeabilized with 1% triton X-100. To detect actin polymerization in lamellipodia, HUVEC were stained with rhodamine-phalloidin (Cytoskeketon Inc., Denver, CO). Cells were viewed under a laser confocal spectral microscope imaging system (Leica, TCS SP5; Mannheim, Germany).

### Capillary-like Tube Formation Assay

Capillary-like tube formation assay was performed as described previously with minor modifications [13]. The 96-well plates were coated with 50 µL Matrigel (10 mg/mL) (BD Bioscience Pharmigen, CA, USA) by incubating at 37°C for 1 h. HUVEC were suspended in M200 (Cascade Biologics, Portland, OR, USA) supplemented with 10% FBS and endothelial cell growth supplement, and plated onto a layer of Matrigel at a density of 4×10^4^ cells/well. The plates were then incubated for a further 4 h at 37°C, and capillary-like tube formation was observed with a microscope.

### Viability Assay

Cell viability was estimated by a modified MTT [3-(4,5-dimethyl thiazol-2-yl)-2,5-diphenyl tetrazolium bromide] (Sigma) assay.

### Subcellular Fractionation

The cells were washed with cold PBS and lysed by Dounce homogenizer in lysis buffer (20 mM Tris, pH 8.0, 3 mM MgCl_2_, 1 mM PMSF), and centrifuged at 12,000 g for 30 min at 4°C. The supernatant was collected as the cytosolic fraction. Pellets were washed with cold PBS, and then homogenized in the lysis buffer (Tris 50 mM, pH 7.5, NaCl 150 mM, PMSF 1 mM, NP-40 1%, 0.1% SDS) on ice, and centrifuged at 12,000 g for 30 min at 4°C. The supernatant was collected as the membrane (particulate) fraction. Proteins of cytosolic and membrane fractions were examined for RhoA, RhoB and RhoC by Western blot analyses.

### Statistical Analysis

All data were expressed as the mean value ± s.e.mean. Comparisons were subjected to one way analysis of variance (ANOVA) followed by Fisher’s least significant difference test. Significance was accepted at P<0.05.

## Results

### Effect of Thy-1Over-expression on the Process of Angiogenesis

**Figure 1:**
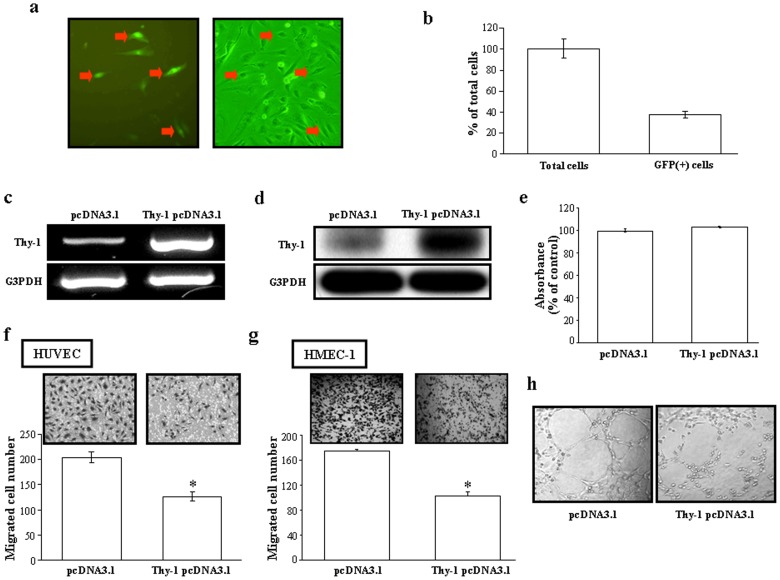
Effects of Thy-1 over-expression on proliferation, migration and capillary-like tube formation in vascular endothelial cells. (a) Representative micrographs of EGFP-positive cells in bright-field image (right panel) and immunofluorescence (left panel) are indicated by the arrows. (b) Quantitative results of the HUVEC expressing Thy-1-pCMS-EGFP-C1 (n = 3). The transfection efficiency of Thy-1-positive cells is around 40%. Transfection of the HUVEC with Thy-1-pcDNA3.1 caused an increase of the levels of Thy-1 mRNA (c) and protein (d) as detected by RT-PCR and Western blot analyses, respectively. For Western blot analysis, membrane was probed with anti-G3PDH antibody to verify equivalent sample loading. RT-PCR products of G3PDH were used as an internal control for RT-PCR analyses. (e) Over-expression of Thy-1 did not affect the proliferation of HUVEC. The cells were transfected with pcDNA301 (control) or Thy-1 pcDNA3.1 for 96 h and then harvested for MTT assay. Values represent the means±s.e.mean. (n = 5). Over-expression of Thy-1 inhibited the migration of HUVEC (f) and HMEC-1 (g). Top panel: a representative result of the migrated HUVEC transfected with pcDNA301 or Thy-1 pcDNA3.1. Bottom panel: quantification of migration inhibition. The quantified results were expressed by percentage of control. Values represent the means±s.e.mean. (n = 3). (h) Over-expression of Thy-1 inhibited the capillary-like tube formation.

**Figure 2:**
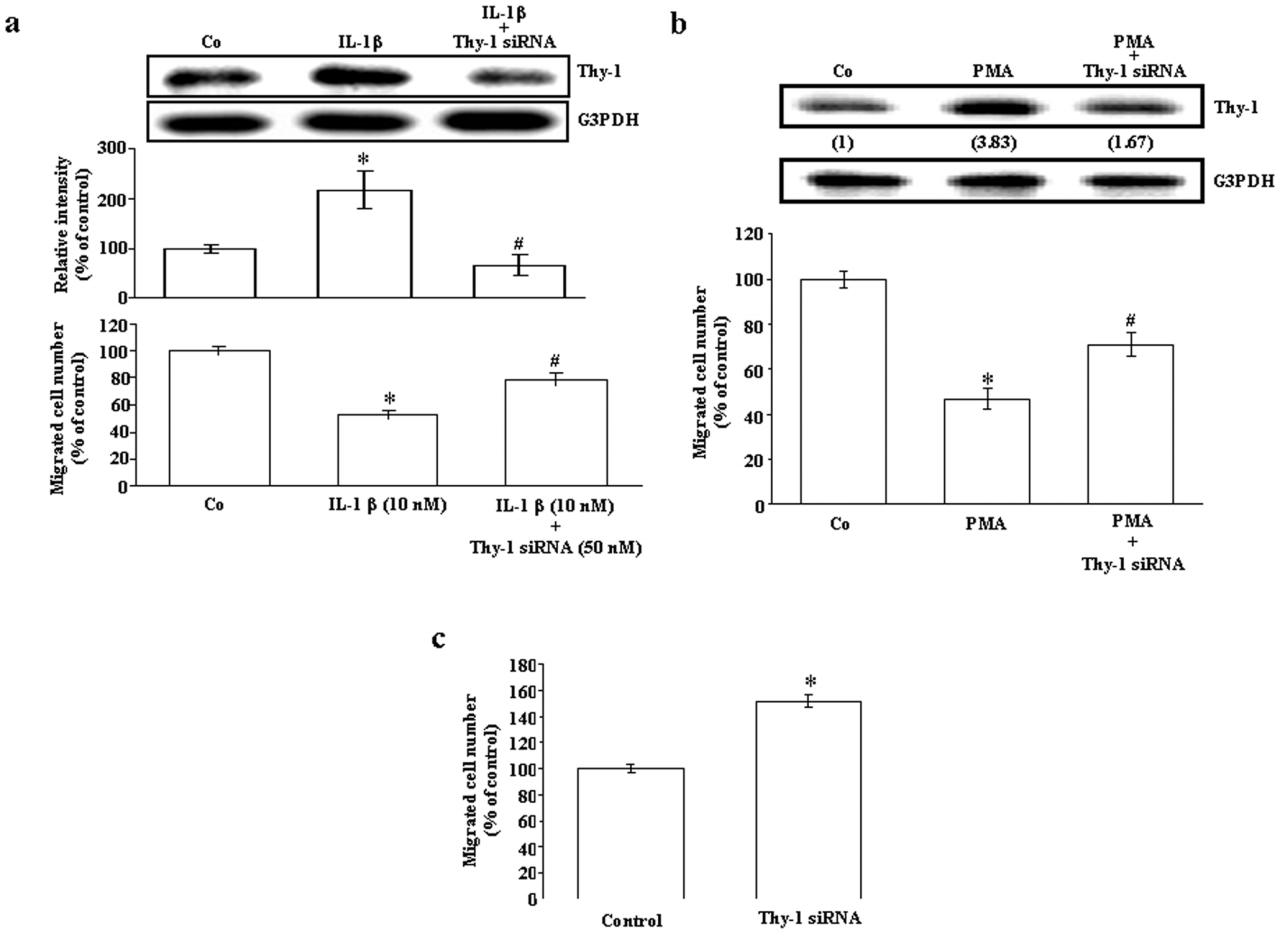
Role of Thy-1 in HUVEC migration. (a) Treatment of HUVEC with IL-1β (10 nM) for 24 h significantly increased the level of Thy-1 protein and decreased the migrated cell number. Values represent the means±s.e.mean. (n = 4). *p<0.05 different from control group. ^#^ *p*<0.05 different from the IL-1β-treated group. (b) Treatment of HUVEC with PMA (20 ng/mL) for 24 h caused a 3.83-fold increase of the Thy-1 protein level (upper panel) and a 54% reduction of the migrated cell number (bottom panel). Values represent the means±s.e.mean. (n = 4). *p<0.05 different from control group. ^#^ *p*<0.05 different from the PMA-treated group. (c) Transfection with Thy-1 siRNA increased the HUVEC migration. Values represent the means±s.e.mean. (n = 3). *p<0.05. Co, control.

To study the role of Thy-1 in the process of angiogenesis, we transfected and over-expressed Thy-1 in HUVEC. Figures 1a and 1b show that the transfection efficiency of Thy-1-positive cells was approximately 40%. The levels of Thy-1 mRNA (Figure 1c) and protein (Figure 1d) were significantly increased in the Thy-1-transfected HUVEC as compared with vector-transfected cells. We used these Thy-1 over-expressed HUVEC to study the role of Thy-1 in the process of angiogenesis. Initially, we examined the effect of Thy-1 over-expression on the proliferation and the migration, two major events during the process of angiogenesis, in HUVEC. To examine whether over-expression of Thy-1 could affect the proliferation of HUVEC, cell number count was performed. As shown in Figure 1e, over-expression Thy-1 for 92 h did not affect the number of HUVEC. We next examined whether over-expression of Thy-1 could affect the endothelial cell migration. As illustrated in Figure 1f, transfection with Thy-1 significantly inhibited the migration of HUVEC. To confirm the effect of over-expression of Thy-1 on the endothelial cell migration, we conducted a similar study in HMEC-1. As shown in Figure 1g, transfection with Thy-1 pcDNA 3.1 also significantly inhibited the migration of HMEC-1. Since tube formation is also involved in the process of angiogenesis, we further examined the effect of Thy-1 over-expression on capillary-like tube formation. As shown in Figure 1h, over-expression of Thy-1 in HUVEC interrupted the capillary-like tube formation. To confirm the role of Thy-1 in endothelial cell migration, the following three experiments were conducted. Initially, we applied the chemicals, IL-1β and PMA, which have been demonstrated to be able to up-regulate Thy-1 expression in vascular endothelial cells, to examine whether over-expression of Thy-1 is correlated with decreased cell migration ability. The results show that treatment with IL-1β at a concentration of 10 nM (Figure 2a, top panel) or PMA at a concentration of 20 ng/mL increased the levels of Thy-1 protein (Figure 2b, top panel) and decreased the number of migrated endothelial cells (Figures 2a and 2b, bottom panel). The IL-1β- and PMA-induced increases of the Thy-1 protein level and decreases of the migrated cell number were prevented by pre-transfection of HUVEC with Thy-1 siRNA (Figures 2a and 2b). Moreover, suppression of endogenous Thy-1 expression by Thy-1 siRNA transfection induced an increase in the migrated HUVEC cell number (Figure 2c).

### Effect of Thy-1 Expression on Lamellapodia Formation, Cell Survival, and Adhesion of Endothelial Cells

**Figure 3:**
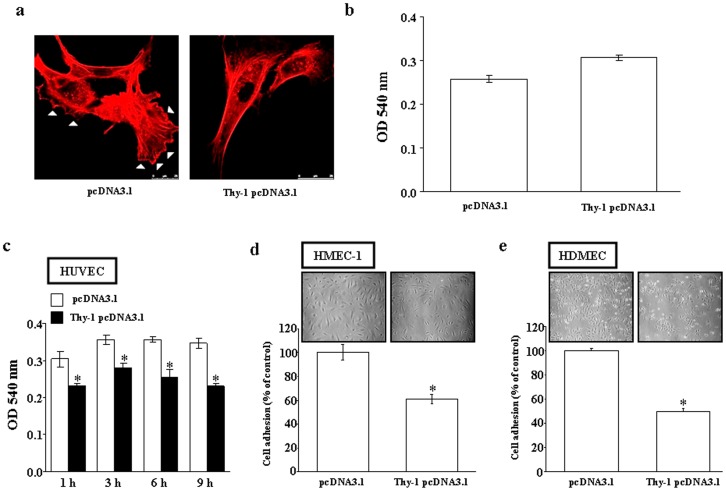
Effect of Thy-1 on lamellipodia formation, and endothelial cell viability and adhesion. (a) Over-expression of Thy-1 inhibited the formation of lamellipodia in HUVEC. HUVEC were tranfected with pcDNA3.1 (control) or Thy-1 pcDNA3.1. At 45 h after transfection, phalloidin staining of HUVEC was then performed as described in the Materials and methods. Scale bar, 25 mm. Triangles indicated the lamellipodia. (b) Over-expression of Thy-1 did not affect the viability of HUVEC. The adhesion of Thy-1-transfected HUVEC to collagen was decreased as compared with vector-transfected HUVEC examined by MTT assay (c) or by cell count (d). (e) This effect was also observed in the HMEC-1 transfected with Thy-1 pcDNA3.1. Values represent the means±s.e.mean. (n = 4–6). *p<0.05.

Since lamellapodia formation, a cytoskeletal protein actin projection on the mobile edge of the cell, is necessary for cell migration, the effect of Thy-1 on lamellapodia formation was also examined. As illustrated in Figure 3a, over-expression of Thy-1 reduced the formation of both stress fibers and lamellipodia in HUVEC. To confirm that the Thy-1-induced decrease in the migration of HUVEC was not due to cell death, we conducted viability assays by comparing the survival rate between vector-transfected and Thy-1-transfected cells at 24 h after transfection. MTT assays showed that there was no significant difference in cell viability between vector-transfected and Thy-1-transfected HUVEC (Figure 3b). Since the attachment and adhesion of endothelial cells to ECM are critical steps for the process of angiogenesis and would affect the endothelial cells invasion and migration, we further examined whether over-expression of Thy-1 could affect the cell adhesion on collagen. The result shows that over-expression of Thy-1 in HUVEC decreased cell attachment on the plate coated with collagen examined by MTT assay (Figure 3c) or by cell count (Figure 3d). We also tested this effect on HMEC-1. As shown in Figure 3e, over-expression of Thy-1 in HMEC-1 also decreased its adhesion on the collagen-coated plate examined by MTT assay.

### Involvement of RhoA Inhibition in the Thy-1-induced Migration Suppression in HUVEC

**Figure 4:**
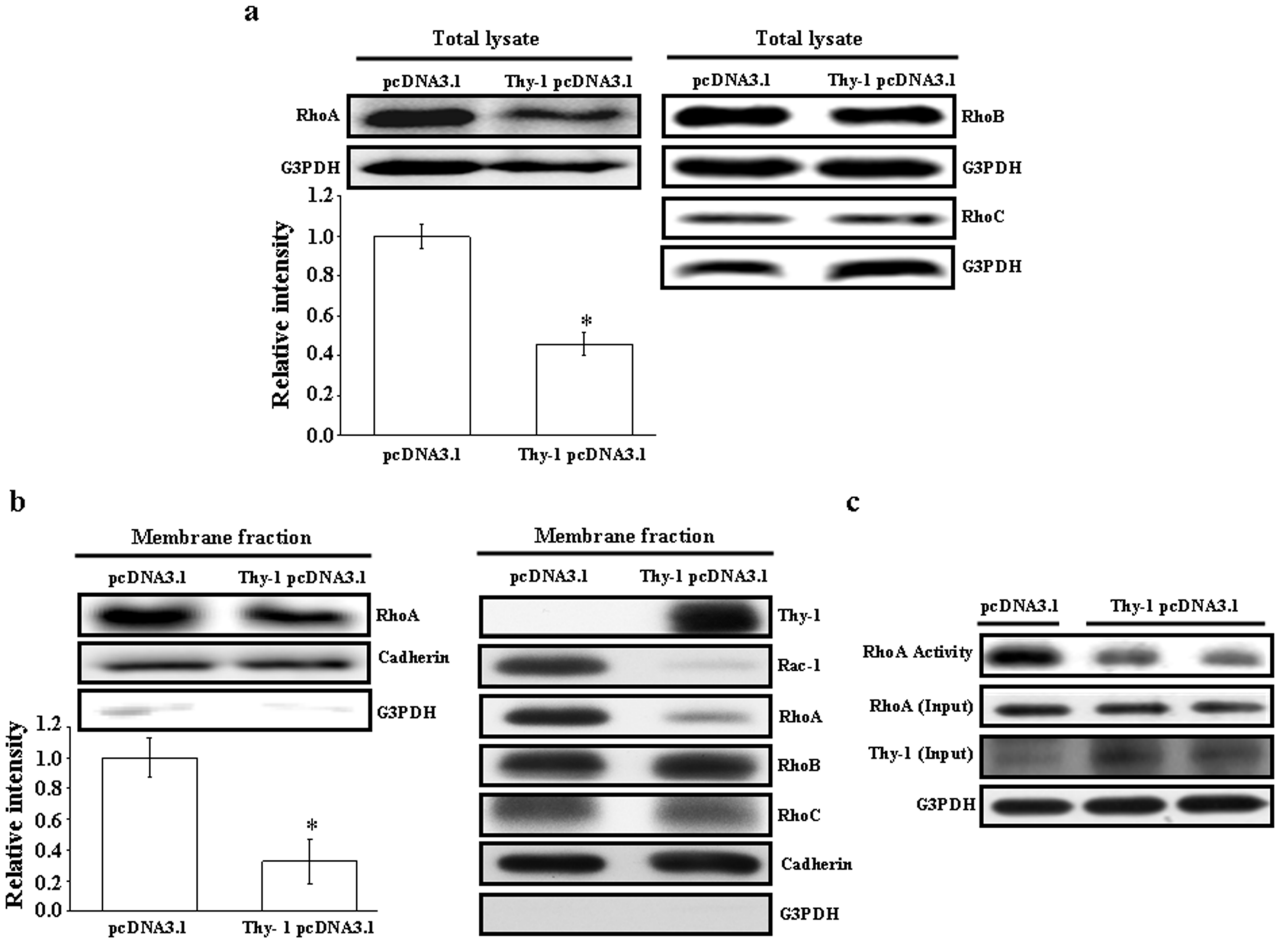
Effects of Thy-1 on expression and activity of RhoA in HUVEC. HUVEC were transfected with Thy-1 pcDNA3.1 or pcDNA3.1 (control). (a) Over-expression of Thy-1 inhibited the total protein levels of RhoA. The bottom panel shows the quantified results after adjusted with their own total protein levels and expressed by fold of control (left panel). Values represent the means ± s.e.mean. (n = 3). *p<0.05. In contrast, the total protein levels of RhoB and RhoC were not significantly affected by over-expression of Thy-1 (right panel). (b) Over-expression of Thy-1 suppressed membrane-bound RhoA. Proteins were detected for RhoA, cadherin and G3PDH by Western blot analysis. Cadherin was used as a cell membrane protein marker to confirm the purities of isolation. The bottom panel shows the quantified results after adjusted with their own total protein levels and expressed by fold of control (left panel). Values represent the means ± s.e.mean. (n = 3). *p<0.05. The protein levels of membrane-bound Rac-1, but not RhoB and RhoC, were also decreased by over-expression of Thy-1 (right panel). (c) A reduced RhoA activity was observed in the HUVEC transfected with Thy-1 pcDNA3.1 as compared with transfected with pcDNA3.1 (control vector).

To delineate the molecular mechanism underlying Thy-1-induced migration inhibition in HUVEC, we examined the protein levels of Rho in the Thy-1-transfected HUVEC. Western blot analysis demonstrated that the protein levels of RhoA (Figure 4a, left panel), but not RhoB and RhoC (Figure 4a, right panel), were significantly decreased in the Thy-1-transfected HUVEC as compared with the control vector-transfected cells. Since translocation of Rho GTPases from the cytosol to the cytoplasmic membrane is required for their activations and functions, we further examined the effect of over-expression Thy-1 on membrane translocation of Rho GTPases. As illustrated in Figure 4b, over-expression of Thy-1 decreased the protein levels of RhoA and Rac-1, but not RhoB and RhoC, in the membrane fraction of HUVEC. To confirm the inhibitory effect of Thy-1 over-expression on the activation of RhoA, the RhoA-GTP pull down assay was conducted. As shown in Figure 4c, over-expression of Thy-1 reduced the RhoA activity in HUVEC.

**Figure 5:**
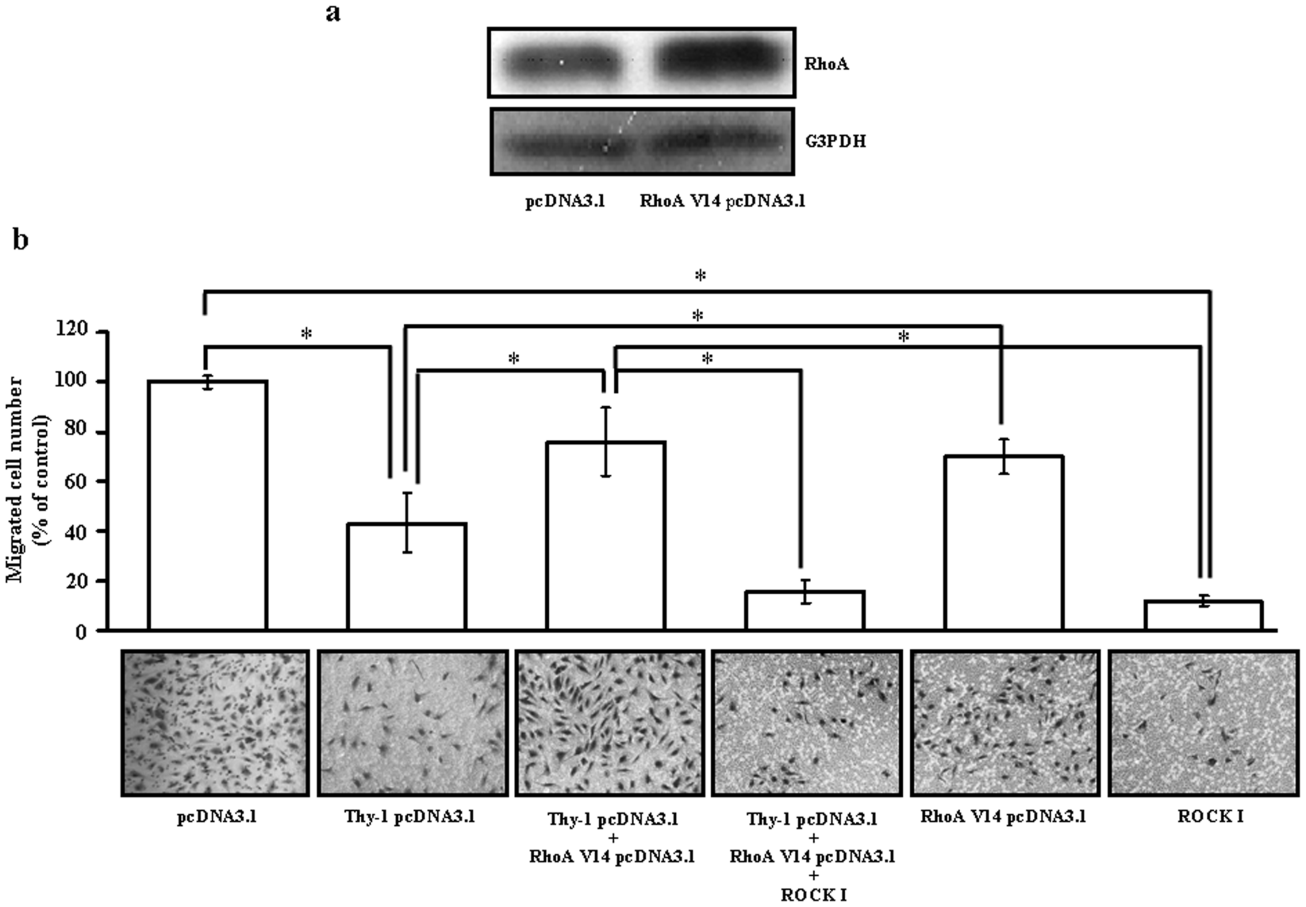
Involvement of the Rho/ROCK signaling pathway in the Thy-1-induced migration inhibition in HUVEC. (a) Tranfection of HUVEC with RhoA V14 increased the expression of RhoA protein in HUVEC. (b) Top panel: over-expression of Thy-1 inhibited the HUVEC migration. However, co-transfection of Thy-1-transfected HUVEC with RhoA V14 prevented the Thy-1-induced migration inhibition. Treatment with 5 µM Y-27632, a ROCK (a kinase associated with RhoA for transducing RhoA signaling) inhibitor, inhibited HUVEC migration and abolished the RhoA V14-induced prevention effect on Thy-1-mediated inhibition of HUVEC migration. Bottom panel: representative photographs demonstrated the results of Figure 5b. Values represent the means ± s.e.mean. (n = 3). *p<0.05.

To further confirm that reduction of RhoA is associated with the Thy-1-induced inhibition of migration activity in the HUVEC, we over-expressed the constitutively active RhoA by transfection of the cells with RhoA V14. As shown in Figure 5a, transfection of HUVEC with RhoA V14 increased the protein levels of RhoA. Figure 5b shows that over-expression of RhoA V14 reduced the degree of Thy-1-induced inhibition of migration activity in the HUVEC. However, transfection of the HUVEC with Thy-1 and RhoA V14 followed by Y-27632 (5 µM), a ROCK inhibitor (a kinase associated with RhoA for transducing RhoA signaling), caused a further suppression of migration activity of the HUVEC.

### Involvement of RhoA Inhibition in the Thy-1-induced Suppression in Capillary-like Tube Formation

**Figure 6:**
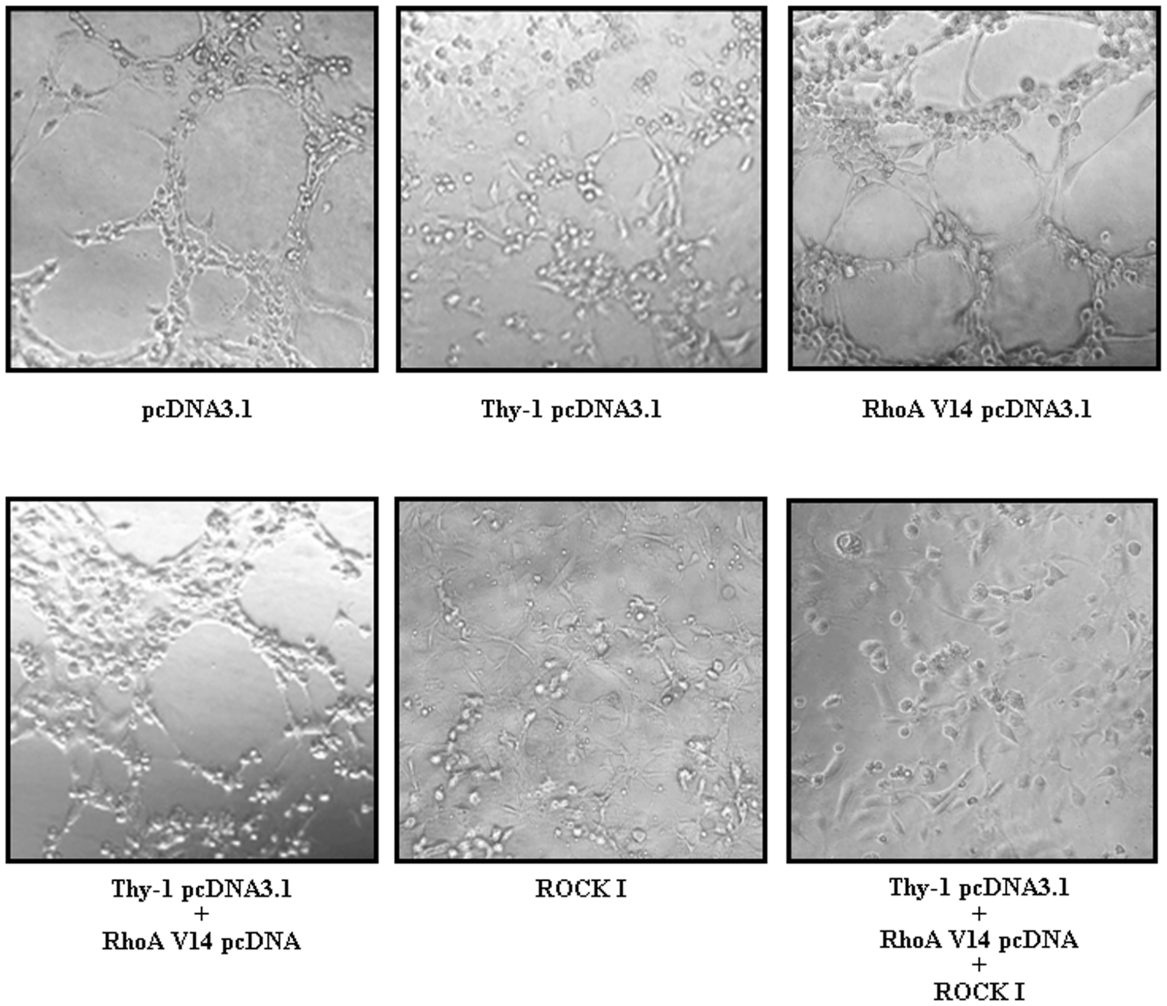
Involvement of Rho/ROCK signaling pathway in the Thy-1-induced inhibition in capillary-like tube formation. Over-expression of Thy-1 inhibited the capillary-like tube formation. However, co-transfection of Thy-1-transfected HUVEC with RhoA V14 prevented the Thy-1-induced inhibition of capillary-like tube formation. Treatment with 5 µM Y-27632 inhibited capillary-like formation and abolished the RhoA V14-induced prevention effect on Thy-1-mediated inhibition of capillary-like tube formation.

We further examined whether the reduction of RhoA also affected the capillary-like formation. As shown in Figure 6, over-expression of Thy-1 inhibited the capillary-like tube formation. Transfection of HUVEC with RhoA V14 abolished the Thy-1-induced inhibition of capillary-like tube formation. However, transfection of the HUVEC with Thy-1 cDNA and RhoA V14 cDNA followed by Y-27632 (5 µM) treatment caused a further suppression of the capillary-like tube formation.

## Discussion

Angiogenesis is a complex multi-step process involving extensive interplay between cells, soluble factors, and ECM components. The processes of angiogenesis involve four distinct sequential steps, including (1) proteolytic breakdown of the basement membrane [14], [15], (2) endothelial cell proliferation, (3) migration of endothelial cells toward the angiogenic stimulus, and (4) lumen formation [2]. Angiogenesis is under tight control by a balance of angiogenesis inducers and inhibitors.

Previously, we have proposed that Thy-1 serves as a marker for angiogenesis and demonstrated that Thy-1 was expressed during physiological and pathological angiogenesis in adult rats, but not during embryonic angiogenesis [4]. Although Thy-1 expression in human endothelial cells has been linked to the induced secretion of matrix metalloproteinase-9 and CXCL8 from neurophil [16], and suggested to play an important role in inflammatory responses [17], what function Thy-1 could have in angiogenic capillary formation is still unclear. Based on the known properties of Thy-1, we consider that it might operate in the modulation or stimulation of endothelial cell proliferation. Surprisingly, we found that over-expression of Thy-1 did not affect the proliferation of cultured endothelial cells (Figure 1e). Instead our results showed that over-expression of Thy-1 inhibited adhesion (Figures 3c–3e), migration (Figures 1f and 1g), and capillary-like tube formation (Figure 1h) of cultured endothelial cells. To confirm the effect of Thy-1 on vascular endothelial migration, we used IL-1β and PMA to induce Thy-1 expression in HUVEC and examined their effects on endothelial cell migration. Our results demonstrated that the level of Thy-1 protein was increased (Figures 2a and 2b, top panel) and the migration of HUVEC was decreased (Figures 2a and 2b, bottom panel) in both IL-1β-treated and PMA-treated HUVEC as compared with vehicle-treated control cells. Thy-1 siRNA transfection, which knocked down the IL-1β-induced and the PMA-induced increases of the Thy-1 protein level (Figures 2a and 2b), prevented the IL-1β-induced and the PMA-induced migration inhibition in HUVEC (Figures 2a and 2b, bottom panel). Furthermore, HUVEC migration was enhanced when the endogenous Thy-1 was knocked down (Figure 2c). These findings led us to hypothesize that Thy-1 is not only a marker of adult new blood vessels, but also an indicator for newly formed blood vessels in the adult just at the cessation of the angiogenic stimulus. Moreover, the findings of the present study suggest that the Rho-mediated pathway might be involved in the signal transduction leading to the inhibition of migration and the suppression of capillary-like tube formation caused by Thy-1 over-expression in cultured HUVEC. This finding seems to be the first demonstration that Thy-1 inhibits endothelial cell migration and capillary-like tube formation through a Rho-dependent pathway.

Vascular endothelial cell adhesion is one of the principal requirements for cell migration and proliferation. Multiple integrins with distinct combinations of α/β subunits have been recognized at such cell sites [18]. Integrin α1β1 and α2β1 mediate cell adhesion on collagen, which is a critical step in initiating tube formation by endothelial cells [19], whereas recruitment of integrin α5β1, the fibronectin receptor, is required for cell migration in the process of angiogenesis. Thy-1 belongs to the immunoglobulin superfamily and has been indicated to be a regulator of cell-cell and cell-matrix interaction. Integrins are one of the major are the major trans-membrane receptors that mediate dynamic interactions between the actin cytoskeleton and the extracellular matrix (ECM) during cell motility. Integrins are αβ heterodimers with an extracellular domain that binds to ECM and links to the actin cytoskeleton. In general, integrins bind to specific motifs within the matrix protein, and the binding specificity is determined by the extracellular domain of integrins that recognize diverse matrix ligands. Previously, Barker et al. demonstrated that Thy-1 expression in fibroblast promotes focal adhesion and stress fiber formation, and inhibits migration through modulation of p190 RhoGAP and Rho GTPase activity [20]. It has been demonstrated that endothelial cell Thy-1 interacts with αvβ3 on melanoma cells and αXβ2 as well as αMβ2 on leukocytes and promote the migration of these cells [21]–[25]. However, the effect of endothelial cell Thy-1 on the migration of endothelial cells has not yet been studied. In contrast to Barker et al.’s report showing that the over-expression of Thy-1 induced adhesion promotion, which might contribute to migration inhibition in rat lung fibroblasts, our results showed that over-expression of Thy-1 inhibited the adhesion of HUVEC on collagen-coated plates (Figures 3c–3e). Adhesion and protrusion are central features of cell migration. Cells exhibit a biphasic migration-velocity response to increasing adhesion strength with fast migration occurring when the strength of adhesion between the cell and the substrate is neither too strong nor too weak. At low adhesion, contraction pulls weak focal adhesions at both the cell front and rear from the substrate; at high adhesion, contraction can not overcome adhesion at the cell front or rear. Therefore, an optimum is reached at intermediate adhesion. Moreover, an optimum in migration speed is also dependent on substrate concentration. This might explain that our results are contrary to those reported by Barker et al. in fibroblasts, where they found that over-expression of Thy-1 in fibroblasts activates RhoA, increases cell adhesion and decreases cell migration [20].

It has been recognized that the post-translational modification of proteins by the addition of isoprenoids is a key physiological process for facilitating cellular protein-protein interactions and membrane-associated protein trafficking [26]. The results from the present study suggest that over-expression of Thy-1 could suppress the endothelial cell migration through altering the prenylation. Prenylation provides important lipid attachments for the posttranslational modification of many proteins, including small GTP-binding proteins belonging to the family of Ras, Rho, Rap, and Rab GTPases. In general, Rho family proteins, which regulate cell motility, require modification with geranylgeranyl pyrophosphate [27]. Blockade of farnesyl biosynthesis leads to an inhibition in the Ras-mediated regulation of proliferation and migration in primary cultured human cells [28]. Tyrosine phosphorylation of focal adhesion kinase (FAK) triggers downstream signaling events including phosphorylation of paxillin, which is required for the regulation of Rho-family GTPases (Rho, Rac and Cdc42) and Pak (a downstream effector of Rac and Cdc42) [29]. To be functionally active, Rho proteins must be localized to the cell membrane by posttranslational modification through addition of isoprenyl groups from isoprenoid pyrophosphate substrates [30]. Rho GTPases play an important role in growth factor-stimulated cell migration and cytoskeletal organization, membrane trafficking, cell cycle control, and transcriptional activation [31]–[34]. To test whether Thy-1-induced HUVEC migration inhibition was mediated through regulating polyisoprenyl pyrophophates synthesis, we examined the effect of over-expression of Thy-1 on the expression of isoprenylated protein. Over-expression of Thy-1 significantly decreased the protein levels of Rho A (Figure 4a, left panel), but not RhoB and RhoC protein (Figure 4a, right panel). Moreover, over-expression of Thy-1 decreased the levels of membrane-bound RhoA and Rac-1, but not RhoB and RhoC (Figure 4b). Figure 4c showed that the RhoA activity was lower in HUVEC transfected with Thy-1 pcDNA3.1 as compared with transfected with pcDNA3.1 (control vector). These data suggested that Thy-1 might interfere with cell migration via suppressing the prenylation of RhoA and Rac-1. This notion was supported by our data showing that over-expression of Thy-1 reduced the formation of stress fibers and lamellipodia in HUVEC (Figure 3a). It has been indicated that the lamellipodia is generally associated with Rac activation, whereas the stress fibers are with Rho activation [35]. Our data showed that over-expression inhibited the activation of both RhoA (Figure 4b, left panel and Figure 4c) and Rac-1 (Figure 4b, right panel) in HUVEC. These data confirm that the formation of stress fibers and lamellipodia in HUVEC was reduced by overexpression of Thy-1.The role of Rho-mediated pathway in Thy-1-induced inhibition of endothelial cell migration was further confirmed by the evidence that (a) over-expression of constitutive active RhoA V14 prevented the migration inhibition caused by over-expression of Thy-1 in HUVEC and (b) pretreatment of the cells with ROCK inhibitor abolished the prevention effect induced by over-expression of RhoA V14 (Figure 5b). Although the difference of migrated cell number between control group (118.89±6.02) and Thy-1 pcDNA3.1+RhoA V14 pcDNA3.1 group (90.28±24.94) did not reach statistical significance (*p* = 0.056), the migrated cell number in the Thy-1 pcDNA3.1+RhoA V14 pcDNA3.1 group is only 76% of the control group. The reduced Rac-1 activity might account for this 24% difference. Our present data suggest that inhibition of RhoA/ROCK signaling is critical for suppressing migration activity in the Thy-1 over-expressed HUVEC. Our results are in agreement with anti-migratory effects in other cells expressing Thy-1, but differ from a previous report showing that Thy-1 inhibited the migration of rat fibroblasts through an increased focal adhesion and Rho GTPase activity [20]. It seems that Thy-1-induced migration inhibition is mediated through different mechanisms in different cell types.

Although our data strongly suggest the important role which Thy-1 might play in endothelial cell migration and capillary-like tube formation, the role of Thy-1 during angiogenesis and the issue of how Thy-1 expression in endothelial cells is regulated are still unclear. Previously, we demonstrated that the expression of Thy-1 in HUVEC is up-regulated by tumor necrosis factor-α and interleukin-1β but not by growth factors, such as basic fibroblast growth factor (bFGF), vascular endothelial growth factor (VEGF), platelet derived growth factor (PDGF), and transforming growth factor-β (TGF-β), suggesting that inflammatory cytokines were probably responsible for this up-regulation of Thy-1 [4]. Moreover, PMA has been shown to be able to induce up-regulation of Thy-1 in human microvascular endothelial cells, suggesting that the protein kinase C pathway might be involved in the regulation of Thy-1 expression in vascular endothelial cells [6]. However, the physiological regulators of Thy-1 expression and the role of Thy-1 during angiogenesis are still unknown and in vivo studies are needed to address these issues.

In conclusion, this study provides the evidence that over-expression of Thy-1 caused inactivation of RhoA, which in turn resulted in the inhibition of endothelial cell migration and capillary-like tube formation. These findings suggest that expression of Thy-1 in endothelial cells during pathological angiogenesis might be important for angiogenesis.
